# Fabrication and Optimization of a Nanoporous Platinum Electrode and a Non-enzymatic Glucose Micro-sensor on Silicon

**DOI:** 10.3390/s8096154

**Published:** 2008-10-01

**Authors:** Yi-Jae Lee, Dae-Joon Park, Jae-Yeong Park, Younghun Kim

**Affiliations:** 1 Department of Electronic Engineering, Kwangwoon University, 447-1, Wolgye-Dong, Nowon Gu, Seoul, 139-701, Korea. E-Mails: yijae2@hotmail.com (Y.J.L.); dj1982dj@hotmail.com (D.J.P.); 2 Department of Chemical Engineering, Kwangwoon University, 447-1, Wolgye-Dong, Nowon-Gu, Seoul, 139-701, Korea. E-Mail: korea1@kw.ac.kr (Y.H.K.)

**Keywords:** Nanoporous platinum, silicon CMOS integrable, non-enzymatic, glucose sensor

## Abstract

In this paper, optimal conditions for fabrication of nanoporous platinum (Pt) were investigated in order to use it as a sensitive sensing electrode for silicon CMOS integrable non-enzymatic glucose micro-sensor applications. Applied charges, voltages, and temperatures were varied during the electroplating of Pt into the formed nonionic surfactant C_16_EO_8_ nano-scaled molds in order to fabricate nanoporous Pt electrodes with large surface roughness factor (RF), uniformity, and reproducibility. The fabricated nanoporous Pt electrodes were characterized using atomic force microscopy (AFM) and electrochemical cyclic voltammograms. Optimal electroplating conditions were determined to be an applied charge of 35 mC/mm^2^, a voltage of -0.12 V, and a temperature of 25 °C, respectively. The optimized nanoporous Pt electrode had an electrochemical RF of 375 and excellent reproducibility. The optimized nanoporous Pt electrode was applied to fabricate non-enzymatic glucose micro-sensor with three electrode systems. The fabricated sensor had a size of 3 mm × 3 mm, air gap of 10 μm, working electrode (WE) area of 4.4 mm^2^, and sensitivity of 37.5 μA•L/mmol•cm^2^. In addition, it showed large detection range from 0.05 to 30 mmolL^-1^ and stable recovery responsive to the step changes in glucose concentration.

## 1. Introduction

As the number of diabetics has increased, many sensitive and selective amperometric glucose sensors to accurately or continuously measure blood glucose levels have been investigated [1-3]. Generally, amperometric glucose sensors are categorized as enzymatic [4] and non-enzymatic ones [5-7]. Most enzyme based sensors utilize glucose oxidase (GOx), which oxidizes glucose and produces hydrogen peroxide. The hydrogen peroxide is measured electrochemically and quantitatively to indirectly determine the amount of glucose. Thus, much research on hydrogen peroxide catalytic electrode based biosensors has been performed [8-10]. The most common and serious problem of enzyme based sensors is a lack of stability, because of the intrinsic nature of the enzyme, while non-enzymatic glucose sensors have many advantages such as stability, simplicity, reproducibility, and no oxygen limitation [21]. Most non-enzymatic and amperometric glucose sensors measure glucose levels by monitoring the current response of glucose oxidation directly at their electrode surfaces [11]. For the direct electro-oxidation of glucose, conventional Pt, Au, and modified Au electrodes [5, 7, 12] were investigated. However, these electrodes have some drawbacks such as low sensitivity and poor selectivity caused by surface poisoning due to adsorbed intermediates and chloride ion [6]. In recent years, another strategy has been pursued in attempts to overcome these problems including the use of porous electrode materials. As examples, nanoporous Pt, nanoparticles and carbon nanotubes have been employed for non-enzymatic and amperometric sensor applications [13-15]. To increase their sensitivities, the RF of the used electrodes should be maximized.

In this paper, a nanoporous Pt on silicon substrate was optimized as a sensitive sensing electrode for silicon CMOS integrable non-enzymatic glucose micro-sensor applications. In order to find a nanoporous Pt electrode with large RF, selectivity and uniformity, optimized electroplating conditions were investigated by varying applied charges, voltages and temperatures. The fabricated nanoporous Pt electrodes were analyzed using AFM and cyclic voltammogrammetry. The nanoporous Pt electrode was then used to fabricate non-enzymatic glucose micro-sensors in order to check its applicability as a sensing electrode.

## 2. Experimental Procedure

### 2.1. Chemicals

All chemicals used were analytical reagent grade. All solutions were prepared with deionized water (resistivity ≥ 18 MΩ-cm). The electroplating mixture for nanoporous Pt was comprised of 42 % (w/w) C_16_EO_8_ (octaethylene glycol monohexadecyl ether, 98 % purity, Fluka), 29 % (w/w) deionized water (18 MΩ-cm) and 29 % (w/w) HCPA (hexachloroplatinic acid hydrate, 99.9 % purity, Aldrich) [16].

The fabricated nanoporous Pt was measured to check its surface RF in 1 molL^-1^ sulfuric acid (H_2_SO_4_, 95-98 %, ACS, Sigma-Aldrich) solution using cyclic voltammograms. The ascorbic acid (AA, 98%, Sigma) and acetaminophen (AP, 99%, Sigma) solutions were prepared by diluting them in a 0.1 mmolL^-1^ phosphate buffered saline (PBS, 0.1 molL^-1^, pH 7.4) solution. The β-D(+) glucose (99.5%, Sigma) stock solution was prepared by diluting it in a 0.1 molL^-1^ PBS (pH 7.4) solution and allowing it to stand for 24 hours before use, in order to allow equilibration. All other electrochemical measurements of the fabricated nanoporous Pt electrode were performed in a three-electrode system by using an electrochemical analyzer (Model 600B series, CH Instruments Inc., USA). A flat Pt bar and an Ag/AgCl electrode with 3 molL^-1^ NaCl were utilized as counter electrode (CE) and reference electrode (RE), respectively.

### 2.2. Design and Fabrication

**Figure 1.**
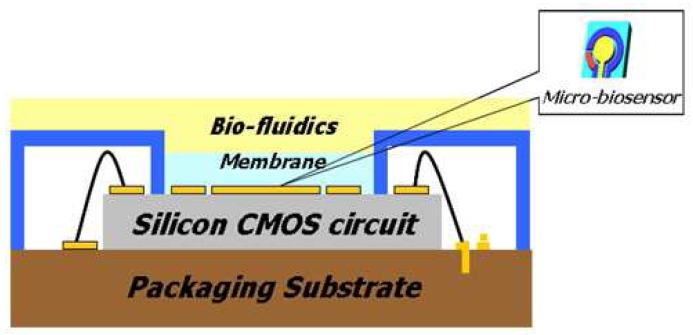
A conceptual drawing of the silicon CMOS integrable non-enzymatic micro-biosensor.

Figure 1 shows a conceptual drawing of a silicon CMOS (complementary metal oxide semiconductor) integrable non-enzymatic micro-biosensor. The silicon CMOS integrable micro-sensors have many preferable advantages such as low cost, small size/volume, mass production, low power consumption, and implantable devices.

**Figure 2.**
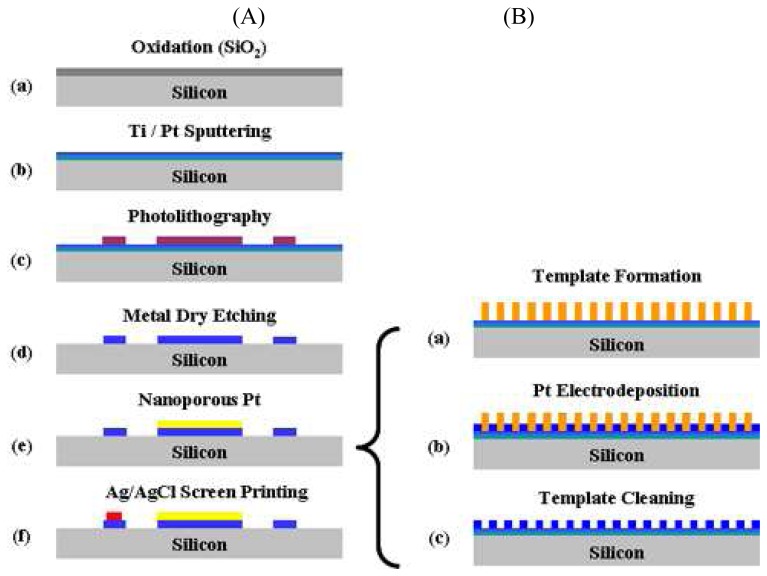
Fabrication procedures of non-enzymatic glucose micro-sensor with nanoporous Pt WE (**A**) and nanoporous Pt electrode (**B**) on silicon substrate.

The proposed non-enzymatic glucose micro-sensor was fabricated as shown in Figure 2(A). Firstly, an insulation layer was deposited on top of the silicon substrate. After sputtering Ti/Pt film on top of the SiO_2_ layer, it was patterned to form three different electrodes (working, counter, and reference electrodes) using the dry-etching technique. Nanoporous Pt was then electroplated on top of the Pt WE [Figure 2(B)]. Liquid crystal templates of C_16_EO_8_ were formed on top of the Pt layer by lowering the temperature down from 85 °C to various temperatures after inserting the sample into the C_16_EO_8_ mixture. At this step, a liquid crystalline hexagonal structure was formed on top of the flat Pt electrode. Pt ions were electroplated into the formed mold by applying a potential between -0.12 and -0.36 V vs. Ag/AgCl [17, 18]. The nanoporous Pt electrode was finally formed by removing the surfactant mold at deionized water. The micro-sensor was finally fabricated by screen printing Ag/AgCl paste on top of the RE.

## 3. Experimental Results and Discussions

### 3.1. AFM analysis

**Figure 3.**
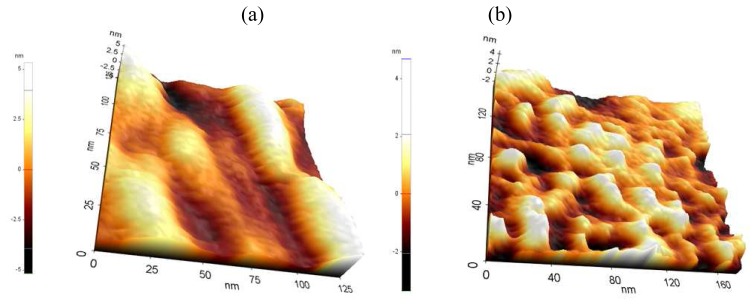
AFM images of fabricated nanoporous Pt at charge at 35 mC/mm^2^ (**a**) and 65 mC/mm^2^ (**b**), respectively.

The surface morphologies of the fabricated nanoporous Pt electrodes were analyzed by AFM (atomic force microscopy, XE-100, Park Systems, Korea). As shown in Figure 3, the surface of the nanoporous Pt electrode was changed by the charge applied during the electroplating process.

While the surface in Figure 3(a) was formed with much smaller Pt grain than Figure 3(b), the surface in Figure 3(b) had many bounding areas, since it was formed with bigger Pt grain than Figure 3(a). Thus, fabricated nanoporous Pt at 35 mC/mm^2^ had a rougher surface as compared to one fabricated at 65 mC/mm^2^. RMS (root mean square) values, which reveal the surface roughness, in Figures 3(a) and 3(b) were 3.041 and 2.291 nm, respectively. In addition, the grain size of the nanoporous Pt electrode fabricated at 65 mC/mm^2^ was smaller than the one fabricated at 35 mC/mm^2^. It should be noted that surface roughness could be adjusted by controlling the applied charge.

### 3.2. Electrochemical analysis

#### 3.2.1 Optimization of nanoporous Pt electrode

**Figure 4.**
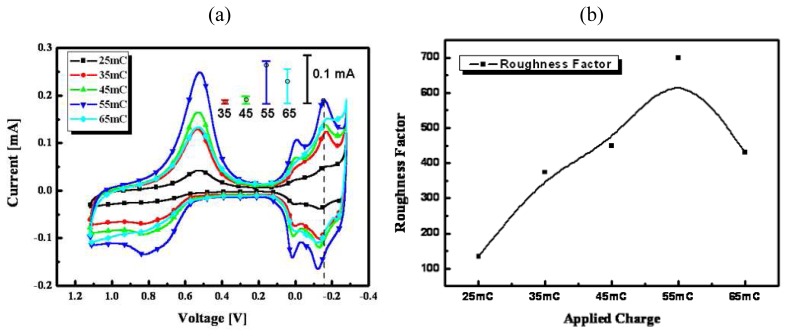
Cyclic voltammograms of nanoporous Pt electrodes electroplated between 25 and 65 mC/mm^2^ of applied charges at the conditions of a fixed voltage of -0.12 V and temperature of 25 °C in 1 mol L^-1^ sulfuric acid (**a**) and their electrochemical RFs (**b**).

The electrochemical surface area of the fabricated nanoporous Pt electrode was determined by cyclic voltammetry in 1 molL^-1^ sulfuric acid solution. Figure 4(a) shows the cyclic voltammogram obtained at scan rate 20 mV/s in the potential range from -0.28 to 1.2 V vs. Ag/AgCl. Its electrochemical RF was calculated in the basis of the method proposed by Biegler *et al.* [20]. In the current and voltage (CV) curve, the first cathodic peak was generated by the reduction of oxygen and the followed twin cathodic peak was generated by the reduction of hydrogen. Since the charge was necessary to form a monolayer of adsorbed hydrogen and the WE area was covered by each hydrogen atom, the electrochemical RF could be calculated. The charge associated with a monolayer of hydrogen was commonly taken as 0.21 mC/cm^2^ [20], the RF was determined as the value of the adsorption charge divided by 0.21 mC/cm^2^.

Figure 4(a) shows cyclic voltammograms in 1 mol L^-1^ sulfuric acid solution of nanoporous Pt electrodes fabricated by electroplating at various applied charges from 25 to 65 mC/mm^2^. In this step, the applied voltage and temperature were fixed at -0.12 V and 25 °C, respectively. The nanoporous Pt electrodes were repetitively fabricated and tested under the respective charge conditions more than five times. The error bars represent current error rate at -0.15 V, which is a bounding point in the CV curve. The current error in these bounding points was obtained by comparing CV curve of the respectively fabricated electrodes. Figure 4(b) shows RFs of fabricated nanoporous Pt electrodes at various applied charges. The respective data points represent the numerical values calculated from the measured cyclic voltammogram (Figure 4(a)). The solid line was made by using interpolation of the calculated roughness data. As shown in Figure 4, the RF of the nanoporous Pt electrode was increased up to 55 mC/mm^2^ as the applied charge increased during the electroplating and the calculated RF at 65 mC/mm^2^ was smaller than that of 55 mC/mm^2^. This might be caused by overplating of Pt into the formed nanomolds. Although the fabricated nanoporous Pt electrode at 45 and 55 mC/mm^2^ had a larger RF than the others, these were not reproducible in repetitive fabrication. Thus, the optimal applied charge was selected as 35 mC/mm^2^.

**Figure 5.**
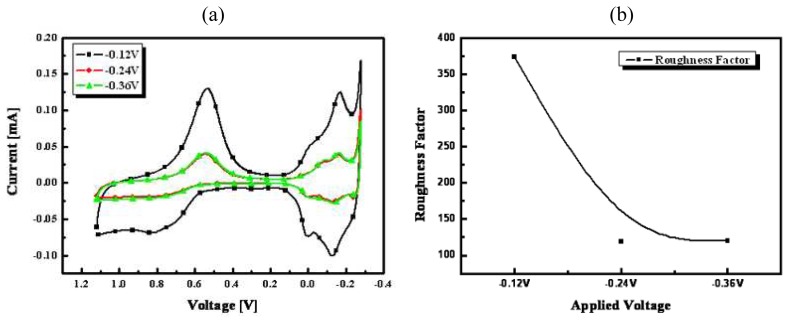
Cyclic voltammograms of nanoporous Pt electroplated between -0.12 and -0.36 mV of applied voltages at the conditions of fixed charge of 35 mC/mm^2^ and temperature of 25 °C in 1 molL^-1^ sulfuric acid (**a**) and their electrochemical RFs (**b**).

Figure 5(a) shows cyclic voltammograms of fabricated nanoporous Pt electrodes at applied voltages from -0.12 to -0.36 mV under conditions of a fixed charge of 35 mC/mm^2^ and a temperature of 25 °C in 1mol L^-1^ sulfuric acid and their calculated and plotted electrochemical RFs [Figure 5(b)]. As shown in Figure 5, RF of nanoporous Pt fabricated at -0.12 V was the largest. Since the formed grain size of Pt was increased as increasing the applied voltage, the Pt was overplated in a short period of time.

**Figure 6.**
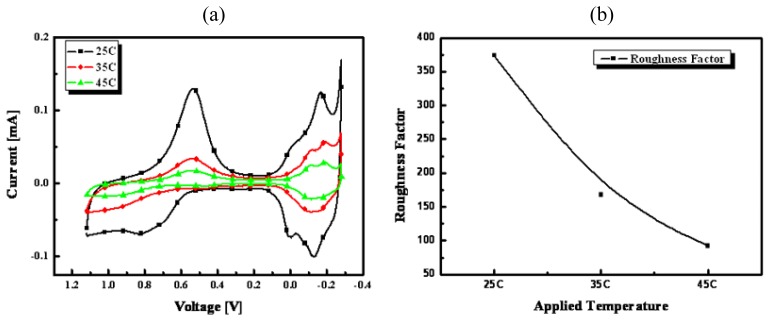
Cyclic voltammograms of nanoporous Pt electroplated between 25 and 45 °C of applied temperature at the conditions of fixed charge of 35 mC/mm^2^ and voltage of -0.12 V in 1 molL^-1^ sulfuric acid (**a**) and their electrochemical RFs (**b**).

Figure 6(a) shows cyclic voltammograms of fabricated nanoporous Pt electrodes at applied temperature from 25 to 45 °C. In this step, applied charge and voltage were fixed at 35 mC/mm^2^ and -0.12 V, respectively. Figure 6(b) shows the RFs of fabricated nanoporous Pt at various applied temperatures. As shown in Figure 6, the RF of nanoporous Pt fabricated at 25 °C was the largest. This might be caused by affecting the formed grain size of Pt, which increased as the applied temperature increased.

#### 3.2.2 Non-enzymatic glucose micro-sensor

**Figure 7.**
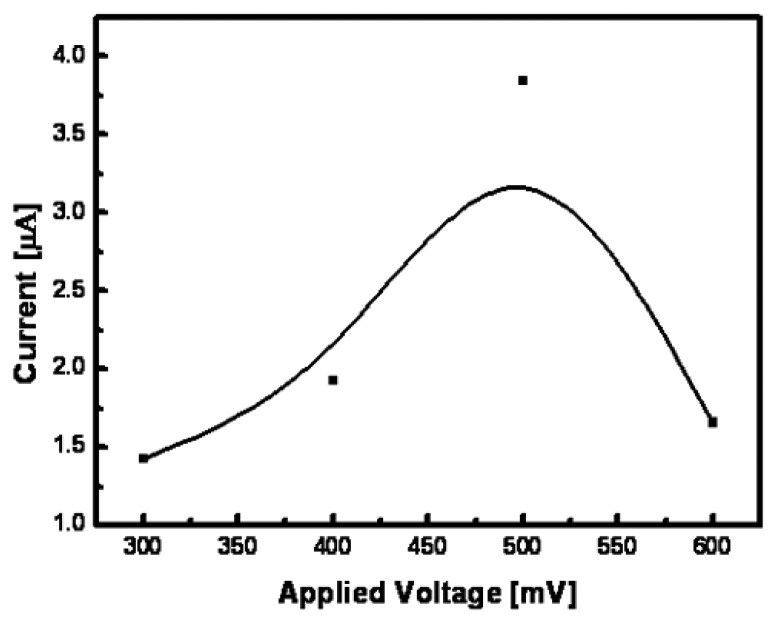
Effects of applied potential on the current response of fabricated non-enzymatic glucose micro-sensor at 1 mmolL^-1^ glucose concentration (pH 7.4).

A non-enzymatic glucose micro-sensor was fabricated to check the applicability of the nanoporous Pt electrode prepared using the optimized fabrication condition. All electrochemical measurements were performed on a three electrodes system by using an electrochemical analyzer (Model 600B series, CH Instruments Inc., USA). Figure 7 shows response currents of the micro-sensor as the applied potential is varied at a glucose concentration of 1mmol L^-1^. As shown in Figure 7, the response current was evidently increased up to 500 mV of applied voltage, which was due to the increment of electron transfer force. However, in order to avoid high working potential causing AA and AP directly oxidized on the electrode surface, the working potential of 0.4 V was selected to be used. The experiments were performed by varying air gap between CE and WE to minimize a size and maximize a sensitivity of non-enzymatic glucose micro-sensor. The fabricated sensors had a size of 3mm × 3mm, WE area of 4.4 mm^2^, and CE width of 300 μm.

**Figure 8.**
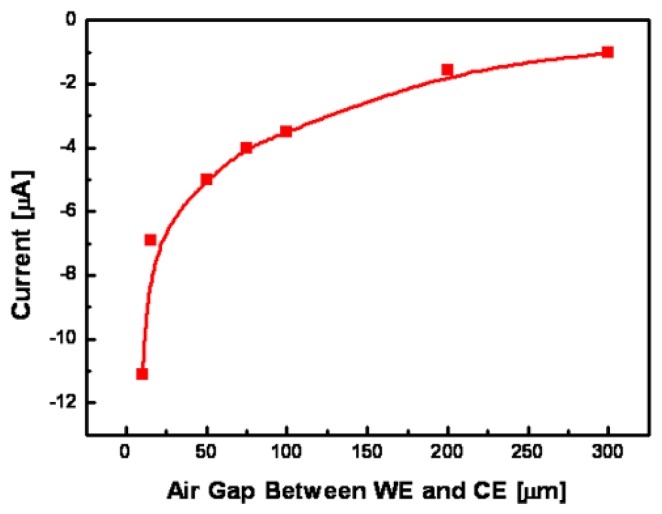
Current responses of non-enzymatic glucose micro-sensors with different air gaps between WE and CE in 5 mmolL^-1^ glucose concentrations (Air gap from 10 to 300 μm).

Figure 8 shows current responses of non-enzymatic glucose micro-sensor with different air gaps (from 10 to 300 μm) between WE and CE at 5 mmolL^-1^ glucose concentrations. The narrower the air gap was between CE and WE, the higher current response obtained was, since the diffusion length of the between WE and CE was shorten for electrochemical activation. However, the micro-sensor with air gap ranged from 3 to 5 μm was not operated, since WE and CE were electrically short.

**Figure 9.**
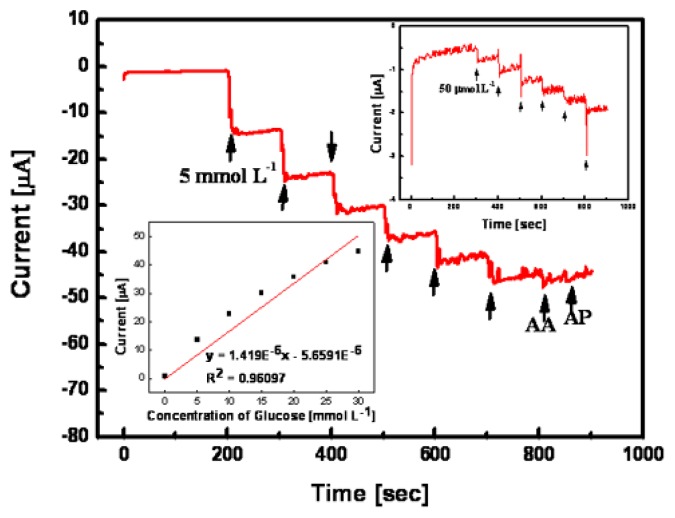
Amperometric current response of optimized non-enzymatic glucose micro-sensor to the consecutive addition of 5 mmolL^-1^ glucose and 0.1 mmolL^-1^ interfering species (AA and AP) in 0.1 molL^-1^ PBS (pH 7.4).

Figure 9 shows amperometric current response of optimized non-enzymatic glucose micro-sensor with nanoporous Pt working electrode to the consecutive addition of 5 mmolL^-1^ glucose and 0.1 mmol L^-1^ interfering species (AA and AP). As shown in Figure 9, the current response of the fabricated sensor was stable and linear without affecting the interfering species. Its sensitivity was 37.5 μA•L/mmol•cm^2^, which was better than that reported in our previous works [18, 19]. In recent reports, the sensitivities of the glucose sensors reached only 17.8 and 11.8 μA•L/mmol•cm^2^ [22, 23]. The upper right trace in Figure 9 shows the current response of the fabricated micro-sensor at an extremely low glucose concentration. As shown in Figure 9, there is no current change at glucose concentrations lower than 0.05 mmolL^-1^. Its glucose detection limit is comparable to previously reported ones [24, 25]. The lower left trace in Figure 9 shows the corresponding calibration curve. Its correlation coefficient was 0.96097. The upward drifting phenomenon at high glucose concentration might be caused by overall noise from the signal.

**Figure 10.**
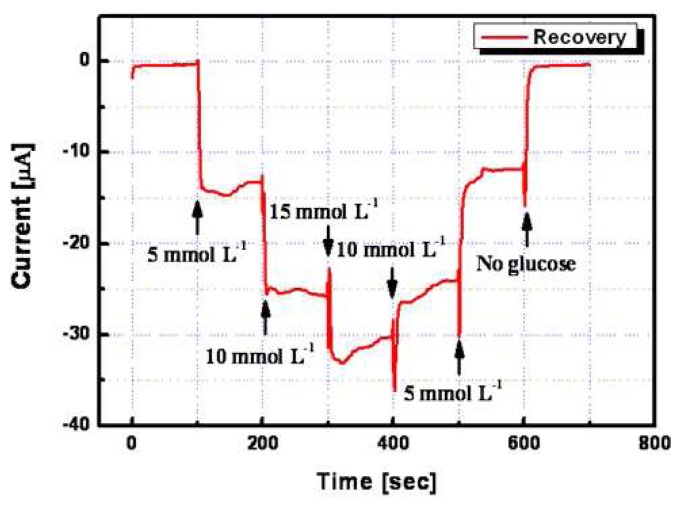
Current–time recordings for recovery characteristic of optimized non-enzymatic glucose micro-sensor in step change of glucose concentration.

Figure 10 shows a current-time recording for the recovery characteristics of the micro-sensor towards stepwise changes in the glucose concentration. This test was performed on different samples of glucose (no glucose, 5, 10, and 15 mmolL^-1^). All measurements were performed by a stepwise change of glucose concentration with 100 sec regular intervals. As shown in Figure 10, its current response was very stable towards both increments and decrements of the glucose concentration. The recovery rates for the no glucose state, 5, and 10 mmolL^-1^ were 102.8 %, 88.7 %, and 93 %, respectively.

## 4. Conclusions

Nanoporous Pt electrodes on silicon substrate have been fabricated and optimized for use as a sensitive sensing electrode for silicon CMOS integrable non-enzymatic glucose micro-sensor applications. The nanoporous Pt electrodes have been fabricated and characterized by varying the applied charges, voltages and temperatures during the electroplating process. By comparison of RFs and reproducibility of nanoporous Pt electrodes, optimal electroplating conditions were determined as an applied charge of 35 mC/mm^2^, a voltage of -0.12 V and a temperature of 25 °C, respectively. When the applied charges, voltages, and temperatures were increased, the reproducibility and RFs of nanoporous Pt were decreased. This might be caused by the rapid increment of Pt grain size and electroplating speed resulting in the overplating of the formed C_16_EO_8_ nanomolds. Non-enzymatic glucose micro-sensors with nanoporous Pt working electrodes have been fabricated and analyzed by varying the air gap between CE and WE. The optimized micro-sensor had a size of 3 mm × 3 mm, an air gap of 10 μm, a CE width of 300 μm, a WE area of 4.4 mm^2^ and a sensitivity of 37.5 μA•L/mmol•cm^2^. The micro-sensor showed a wide detection limit range from 0.05 to 30 mmolL^-1^ and stable recovery response to stepwise changes in the glucose concentration. These results show that the fabricated CMOS integrable non-enzymatic micro-sensors are promising for *in-vitro* and *in-vivo* miniaturized hand held health care system and continuous monitoring system applications.
